# Substance Use, Criminal Recidivism, and Mortality in Criminal Justice Clients: A Comparison between Men and Women

**DOI:** 10.1155/2018/1689637

**Published:** 2018-03-11

**Authors:** Caroline Mannerfelt, Anders Håkansson

**Affiliations:** Psychiatry, Department of Clinical Sciences Lund, Faculty of Medicine, Lund University and Malmö Addiction Center, Skåne Region, Sweden

## Abstract

*Background. *This study aimed to map differences between male and female offenders with substance abuse, with respect to descriptive characteristics and risk factors for mortality and criminal recidivism.* Methods.* Criminal justice clients with substance abuse problems (*n* = 7085) were interviewed with the Addiction Severity Index. Mortality and data on return to criminal justice were retrieved from national registers.* Results. *Female offenders reported heavier substance use patterns, more psychiatric symptoms, and more often a partner with substance abuse, but had lower mortality (2% versus 4%) and criminal recidivism (62% versus 71%) during follow-up. Having a substance-abusing partner was associated with criminal recidivism among females.* Conclusions. *Female offenders with substance abuse differ from their male counterparts. Males and females had different risk factors for criminal recidivism.

## 1. Introduction

Substance abuse is common in criminal justice populations. In a review article from 2006, Fazel et al. estimated the prevalence of drug abuse among male and female prisoners to be 10–48% and 30–60%, respectively, and the prevalence of alcohol abuse or dependence to be 18–30% in the male clients and 10–24% in the female clients, respectively [1]. A recent survey carried out on a national level with the purpose of identifying characteristics of the clients in the Swedish criminal justice system found that 70% of all clients had substance abuse problems (including alcohol, drugs, or both), with a slightly lower percentage among female clients compared to the whole group (67%) [2]. These numbers can be compared to the general population in the same nation, where 7.6% of the men and 4.3% of the women fulfill the criteria for alcohol dependence or abuse and 1.0% of all men and 0.5% of all women fulfill the criteria for drug dependence or abuse [3].

An important task of the criminal justice system is to prevent criminal recidivism. Substance abuse is a strong risk factor for committing new offenses and therefore presents a difficult challenge for correctional institutions [4, 5]. Apart from contributing to criminal relapse, the high prevalence of substance abuse has been shown to be associated with elevated mortality and psychiatric symptoms in the criminal population, compared to the population in general [6, 7].

*Female Offenders with Substance Abuse: A Group with Different Needs?* Historically, substance use disorders addressed in research and policy have been more common among men than among women, although this gap may have become narrower in recent years [8]. Clearly, the male predominance in substance users is even larger in criminal justice settings, also making gender-specific research in this setting more challenging. In the setting assessed in the present study, only seven percent of prisoners are women [9].

In recent years, however, there has been a growing amount of research on female criminal clients with substance abuse [10]. The findings suggest that female clients in the criminal justice system differ from their male counterparts in a number of aspects. Compared to men, women are more often convicted of drug crime and property crime and more rarely of violent crime. Female clients generally report many psychiatric symptoms and have often experienced psychological, physical, or sexual abuse during their lifetimes. These gender-specific findings have led researchers to advocate that female clients may benefit from special interventions, such as individualized treatment and women-only groups [10].

Despite the growing amount of research done on female criminal offenders with substance abuse in recent years, the research gap remains, as many of the studies have been conducted on a female-only population [10, 11]. While these studies have identified important characteristics in the female subpopulation, it is difficult to tell whether women have different risk factors than men, as no comparisons are made.

The aim of this study is to map differences between substance-abusing men and women in the Swedish criminal justice system and to investigate whether male and female offenders have different risk factors associated with mortality and criminal recidivism.

## 2. Methods

### 2.1. Data Material

The material used in this study consists of interview data from the Swedish Prison and Probation Service. In order to evaluate treatment needs, clients with known or suspected substance abuse in the Swedish criminal justice system are interviewed with the Addiction Severity Index (ASI). The ASI is a semistructured interview, first developed in the 1980s, which is used to assess seven different areas of life: demographic information, physical health, employment, substance abuse, criminality and legal problems, family and social relationships, and psychiatric health [12]. The ASI is used commonly in social and clinical work, as well as addiction research [13, 14]. The ASI exists in several different versions, and the version of ASI used at the time of the study in the Swedish criminal justice system was the ASI-X, which is comparable with the original ASI version [15].

The interview material in the present database was collected by the Swedish Prison and Probation Service in a number of correctional facilities between 2001 and 2006. The database contains interview material from 7085 clients, of whom 12 percent are women (*n* = 822) and 88 percent (*n* = 6263) are men. A majority of all clients were interviewed in prison (*n* = 5122), and the rest were interviewed while on probation (*n* = 1189), while on remand (*n* = 386), or in other types of correctional facilities (*n* = 246). Ninety percent of clients were interviewed within the first year from entry to the criminal unit where the interview took place, and 97 percent within two years.

The database was coded by the Swedish Prison and Probation Service and delivered to the research group in 2006. Several previous publications have been based on the whole material, including follow-up analyses of mortality and criminal recidivism [16, 17].

### 2.2. Statistical Analysis

A number of baseline variables were chosen from the ASI, including all the following variables: age (at baseline), urban residence (large city or not), country of origin (Sweden, Denmark, Norway, Finland, or Iceland versus other countries), homelessness 30 days prior to incarceration, whether or not the client has any children, prevalence of a chronic physical disease (defined in the ASI manual as a medical problem requiring continuous or regular care), whether or not the client has worked during the three years prior to incarceration, whether or not the client has a partner with substance abuse, lifetime history of substance abuse (defined as regular use for six months or more) of binge drinking, heroin, methadone, other opioids, sedatives, cocaine, amphetamine, or cannabis, lifetime history of injection drug use, mean number of substances used 30 days prior to incarceration, main crime in index verdict (violent crime, property crime, drug crime, or financial crime), history of psychiatric hospitalization, and lifetime history of psychiatric symptoms (depression, anxiety, cognitive problems, hallucinations, difficulty controlling violent behavior, and previous suicide attempt).

Baseline variables were analyzed for men and women separately in the whole sample, using chi-square analyses for categorical variables and independent sample *t*-tests for continuous variables.

Binary associations between baseline variables and all three outcomes (death status, criminal recidivism, and psychiatric hospitalization) were analyzed in the same way, for men and women, respectively.

The baseline variables which had a significant association (*p* < 0.05) with outcome in the binary associations analyses were entered simultaneously in a Cox regression analysis, for each outcome and sex. The results were presented as hazard ratios with a 95% confidence interval.

All statistical analyses were carried out in SPSS version 22.0. The analyses of the selected outcomes have been approved by the Regional Ethics Review Board, Lund, Sweden (ethical approval number 2009-328).

### 2.3. Outcome Data

In order to assess the three different outcomes, data were collected from external resources and incorporated in the database. The merging of external data to the ASI database was performed by the Swedish Prison and Probation Service on request by the research group, as the ASI database is coded and the research group does not have access to the code key.

Mortality data were collected from the Swedish National Causes of Death Registry, held by the National Board of Health and Welfare [18]. Mortality was defined as death according to the Swedish National Causes of Death Registry. Time at risk for death was measured from the termination of the index sentence, until death, or until data was censored (December 31, 2008).

Data concerning criminal recidivism were collected from the criminal justice registry [17]. Criminal recidivism was defined as any return to the criminal system. Time at risk for returning was measured from the termination of the index sentence, until return to the criminal system, or until data was censored (November 2010), or until time of death.

### 2.4. Inclusion and Exclusion

Interviews from 7085 criminal clients were available in the database. Fifty of these (0.7%) were excluded from the beginning, as the interviewer had perceived the client to lie or misinterpret the interview questions.

The baseline data and mortality analyses contained interview data from all remaining 7035 clients. The analyses of criminal recidivism were conducted on the criminal clients who were interviewed in prison, as the follow-up data were only systematic for those clients (*n* = 5122). Clients were further excluded if their dominating substance abuse problem was “none” (*n* = 720) or missing (*n* = 127), as the study aimed to analyze only criminal justice clients with substantial substance abuse. Clients were also excluded if they had been interviewed with the adolescent version of ASI, the ADAD (Adolescent Drug Abuse Diagnosis, *n* = 5). Out of the remaining clients, analyses were carried out on those who were confirmed to have left prison, that is, clients with a confirmed date of release (*n* = 4081).

## 3. Results

### 3.1. Baseline Variables

The analyses of the chosen baseline variables showed that women had a higher mean age than men and that they to a higher extent were born in Nordic countries, while more men lived in large cities. More female clients had children.

Prevalence of chronic physical disease was higher in the female study population, and more female offenders than male offenders reported having partners with substance abuse.

The analyses of lifetime history of substance abuse showed that amphetamine, methadone, and injection drug use (regardless of which drug) were more common in the female study population, while cocaine and cannabis were more common in the male client population. The mean number of substances used in the last 30 days before incarceration was higher among male clients.

Male clients were convicted of violent crimes and property crimes to a higher extent than female clients, while financial crime was more common in the female population.

Analyses of lifetime history of psychiatric symptoms showed that female clients reported psychiatric hospitalization, suicide attempt, depression, and anxiety more than male clients, while male clients reported more difficulty in controlling violent behavior.

Baseline variables for both sexes are shown in Table 1.

### 3.2. Mortality

A total of 804 female clients were available for the mortality analyses, and two percent (*n* = 16) of the female population died during the follow-up period. A total of 6231 male clients were available for the mortality analyses, and four percent (*n* = 252) of the male population died during the follow-up period.

Binary associations between the baseline variables and mortality status for both sexes are reported in Table 2.

No baseline variable was found to have a significant association (*p* value < 0.05) with mortality status among the female clients. Therefore, no Cox regression analysis was performed.

When entered in a multivariate analysis, three baseline variables produced a positive association with mortality among the male clients: these were age, heroin use, and amphetamine use.

See Table 3.

### 3.3. Criminal Recidivism: Female Clients

A total of 407 female clients were available for follow-up analysis of criminal recidivism, and 62 percent (*n* = 254) of these returned to the criminal justice system during the follow-up period. A total of 3674 male clients were available for the analyses of criminal recidivism, and 71 percent (*n* = 2599) of these returned to the criminal system during the follow-up period.

Binary associations between baseline variables and criminal recidivism for both sexes are shown in Table 4.

The multivariate regression analysis showed that having a partner with substance abuse and having a property crime as the main crime in the index verdict were variables positively associated with criminal recidivism among the female clients. Having a violent crime as the main crime in the index verdict was negatively associated with criminal recidivism.

See Table 5.

The multivariate Cox regression analysis showed that a lifetime history of heroin use, amphetamine use, injection drug use, main crime in index being property crime, and a lifetime difficulty of controlling violent behavior were all positively associated with criminal recidivism in the male population. Residence in a large city, paid work in the last three years before incarceration, and having a violent crime as the main crime in the index verdict were variables negatively associated with criminal recidivism.

See Table 6.

## 4. Discussion

The present study aimed to identify differences between male and female criminal offenders with substance abuse and to investigate whether they have different risk factors for mortality and criminal recidivism after the end of sentence. The analyses of baseline variables showed gender differences similar to those found earlier in the Swedish criminal client population: female clients were older than male clients and reported more physical disease and psychiatric symptoms [2].

Analyses of substance abuse variables showed that cannabis and cocaine use were more common among male offenders, while methadone, amphetamine, and injection drug use were more common among the female offenders. This finding suggests that female offenders have a different and potentially heavier pattern of substance abuse, compared to both male offenders and women with substance abuse in the general population, as women in the general population are less likely to use “hard drugs” than men [3]. In a study on gender differences in drug misuse among arrestees in the UK, Holloway and Bennett showed similarly that female arrestees had a heavier pattern of drug abuse than male arrestees, although the dominating drugs among them differed from those in the present study (heroin and crack cocaine) [19]. Analogous gender differences have been found among prisoners in the US [20].

The high prevalence of psychiatric symptoms among female clients in this study is consistent with another research comparing psychiatric symptoms among male and female prisoners [11]. Also, consistent with the present study, previous follow-up data in prisoners have demonstrated that differences are particularly pronounced for depression and anxiety [9].

Very few women died during the follow-up period, and no significant results were found regarding risk factors for mortality in the female subgroup. Heroin use, amphetamine use, and injection drug use in general were associated with mortality among the male clients. It has been suggested that the elevated mortality among criminal offenders is due to drug overdoses and other drug-related deaths, following prison release. It is interesting to note that female offenders in the present study differ from the male offenders in this aspect, as no substance abuse variables correlated with elevated mortality, in spite of the fact that the female offenders reported a heavier pattern of substance abuse. However, the low number of deaths limits the possibility of obtaining significant results in the present study. Female gender has been found to be negatively associated with death in previous research on mortality among criminal clients [16].

A higher percentage of the male prisoners returned to the criminal justice system during the follow-up period, compared to the female prisoners (70% versus 60%). Substance abuse variables and variables related to substance abuse, like homelessness in the last month prior to incarceration, were associated with criminal recidivism among both the male clients. These results support previous findings that criminal recidivism among criminal clients with substance abuse is directly linked to the severity of the substance abuse [17]. However, no substance abuse variable was associated with criminal recidivism among the female prisoners. The only variables that were associated with elevated risk factors for criminal recidivism in the female study population were main crime in index verdict being a property crime and having a partner with substance abuse. This suggests that female offenders have different factors sustaining a criminal lifestyle than male offenders.

In the baseline comparison, the women in this study reported having partners with substance abuse to a much higher extent than their male counterparts. Previous research on gender differences in drug-use careers has identified that female substance abusers are influenced to use drugs by sexual partners or spouses, as opposed to male substance abusers, who are more influenced by peer pressure [20]. Importantly, in the present study, having a partner with substance abuse was predictive of criminal recidivism. Intuitively, this calls for further focus on the treatment of female offenders' partners, in order to facilitate a favorable outcome in substance abuse treatment and rehabilitation of women. This is consistent with previous findings indicating that the substance use of a male partner has a negative influence on a woman's substance use treatment [21]. Also, in addition to the importance of treatment for the male partner, the present findings also point towards the need for methods aiming to treat an index person with a substance use disorder by means of her concerned significant others, including a partner, with interventions such as the Community Reinforcement and Family Training (CRAFT) model [22].

Substance abuse treatment in a correctional setting serves two main purposes: to treat substance abuse and, as a direct consequence, to prevent criminal recidivism. The present study adds to the growing amount of evidence that female offenders with substance abuse differ in many aspects from both male offenders with substance abuse and women in the general population with substance abuse. More research is warranted regarding successful gender-specific substance abuse treatment for female criminals. The results in the present study indicate that targeting the issue of the female offenders' partners with substance abuse might be an important aspect of gender-specific treatment, as having a partner with substance abuse was found to be a significant risk factor for criminal recidivism in the female subgroup, and this may need to be further investigated further in future studies.

*Strengths and Limitations.* One limitation in this study is that baseline material is based on self-reported data. There is a risk of recall bias, especially regarding the time lapse between entry in the criminal justice system and the time of the ASI interview. The ASI interviewers were trained and supplied with a manual in order to evaluate the answers of the interviewed individuals. A number of interviews from the database were excluded before analysis, as the interviewers had perceived the interviewed individuals to lie, or misinterpret, the interview questions. Despite these efforts, the risk of misinterpretation, recall bias, and other limitations associated with self-reported data cannot be ruled out.

Another limitation is related to statistical power. Women were in minority in the study population, and, as a direct consequence, fewer significant risk factors were found regarding all three outcomes, compared to the male sample. Because of this, the question about whether men and women have different risk factors for mortality and criminal recidivism remains partially unanswered. An ideal study would have included similar numbers of men and women and an appropriate sample size, for correct comparison. However, as women are in minority in the Swedish criminal justice system, a prospective study including large enough study samples of both men and women would have taken many years to compile.

On the other hand, while the female and male subgroup differed in size, the database used in this study consists of a large material. The female subgroup, although smaller than the male subgroup, is still larger than the study populations in many of the previous studies of women with substance abuse in correctional institutions [10, 20].

Another strength of this study is that it is quantitative and that women and men are compared on the same variables. Previous researches on female offenders with substance abuse have often been qualitative and carried out in a small, female-only population, which makes gender comparisons difficult [10].

### 4.1. Conclusions

Female offenders with substance abuse had different substance abuse patterns than male offenders, with more amphetamine and intravenous drug use. More female offenders had a partner with substance abuse. Female offenders also reported psychiatric symptoms to a higher extent than their male counterparts. Substance abuse variables were positively associated with mortality and criminal recidivism for male offenders, but not for female offenders. Female gender was negatively associated with mortality, despite the fact that female offenders reported a heavier pattern of substance abuse. Having a partner with substance abuse was found to be a gender-specific risk factor for criminal recidivism among the female offenders. The present findings suggest further comparative research, conducted on study populations with equal numbers of men and women, and also research investigating whether targeting the partners with substance abuse of the female offenders might be a successful gender-specific treatment strategy.

## Figures and Tables

**Table 1:** Baseline characteristics.

	Men% (*n*)	Women% (*n*)	Total% (*n*)	*p* value
*N* =	6231	804		
Mean age^*∗*^	32.3 years	36.2 years	32.7 years	**<0.001**
Urban residence, large city^*∗*^	45.5 (2838)	39.4 (317)	44.8 (3155)	**0.001**
Country of birth, Nordic country^*∗*^	81.1 (5053)	91.0 (732)	82.2 (5785)	**<0.001**
Homelessness, last 30 days	16.9 (1052)	19.0 (153)	17.1 (1205)	0.128
Client has children^*∗*^	45.3 (2830)	64.8 (521)	47.5 (3341)	**<0.001**
Chronical physical disease^*∗*^	44.8 (2792)	57.1 (459)	46.2 (3251)	**<0.001**
Client has worked for the last 3 yrs	38.9 (2425)	36.1 (290)	38.6 (2715)	0.118
Client has partner with substance abuse^*∗*^	10.0 (620)	34.0 (273)	12.7 (893)	**<0.001**
*Lifetime history of substance abuse (>1 yr)*				
Binge drinking	41.3 (2572)	40.2 (323)	41.2 (2895)	0.550
Heroin	17.5 (1092)	19.3 (155)	17.7 (1247)	0.221
Methadone^*∗*^	2.9 (179)	4.7 (38)	3.1 (217)	**0.004**
Other opioids	12.8 (796)	14.2 (114)	12.9 (910)	0.264
Sedatives	29.0 (1807)	26.7 (215)	28.7 (2022)	0.183
Cocaine^*∗*^	13.7 (854)	5.5 (44)	12.8 (898)	**<0.001**
Amphetamine^*∗*^	50.6 (3154)	54.5 (438)	51.1 (3592)	**0.039**
Cannabis^*∗*^	53.6 (3337)	38.2 (307)	51.8 (3644)	**<0.001**
Injection drug use^*∗*^	47.9 (2984)	54.4 (437)	48.6 (3421)	**0.001**
Mean number of substances used, last 30 days^*∗*^	1.4	1.3		**0.003**
*Main crime in index verdict*				
Violent crime^*∗*^	13.4 (838)	10.1 (81)	13.1 (919)	**0.008**
Property crime^*∗*^	21.9 (1367)	15.4 (124)	21.2 (1491)	**<0.001**
Drug crime	22.9 (1429)	24.8 (199)	23.1 (1628)	0.250
Financial crime^*∗*^	3.8 (238)	6.1 (49)	4.1 (287)	**0.002**
*Lifetime history of psychiatric problems*				
Hospitalization^*∗*^	14.0 (871)	21.8 (175)	14.9 (1046)	**<0.001**
Suicide attempt^*∗*^	19.0 (1185)	35.2 (283)	20.9 (1468)	**<0.001**
Depression^*∗*^	49.3 (3073)	62.7 (504)	50.8 (3577)	**<0.001**
Anxiety^*∗*^	51.3 (3198)	66.7 (536)	53.1 (3734)	**<0.001**
Cognitive problems	51.7 (3219)	52.9 (425)	51.8 (3644)	0.522
Hallucinations	12.7 (793)	13.2 (106)	12.8 (899)	0.715
Difficulty controlling violent behaviour^*∗*^	41.8 (2605)	32.2 (259)	40.7 (2864)	**<0.001**

^*∗*^Variable significantly associated with the outcome variable.

**Table 2:** Characteristics according to mortality status.

	Women			Men		
	Deceased, % (*n*)	Survived, % (*n*)	*p* value	Deceased, % (*n*)	Survived, % (*n*)	*p* value
*N* =	*16*	*788*		*252*	*5979*	
Mean age	39.75 years	36.14 years	0.201	34.90 years	32.20 years	**<0.001**
Urban residence, large city	31.3 (5)	39.6 (312)	0.610	44.8 (113)	45.6 (2725)	0.819
Country of birth, Nordic country	93.8 (15)	89.6 (706)	1.000	86.5 (218)	78.2 (4677)	**0.002**
Homelessness, last 30 days	6.3 (1)	19.3 (152)	0.331	19.4 (49)	16.8 (1003)	0.268
Client has children	75.0 (12)	64.6 (509)	0.443	50.8 (128)	45.0 (2692)	0.071
Chronic physical disease	62.5 (10)	57.0 (449)	0.659	52.8 (133)	44.5 (2659)	**0.009**
Client has worked for the last 3 yrs	31.3 (5)	36.2 (285)	0.685	28.2 (71)	39.4 (2354)	**<0.001**
Client has partner with substance abuse	37.5 (6)	33.9 (267)	0.762	10.3 (26)	9.9 (594)	0.842
*Lifetime history of substance abuse (>1 yr)*						
Binge drinking	62.5 (10)	39.7 (313)	0.066	47.2 (119)	41.0 (2453)	**0.050**
Heroin	37.5 (6)	18.9 (149)	0.062	33.3 (84)	16.9 (1008)	**<0.001**
Methadone	12.5 (2)	4.6 (36)	0.127	4.4 (11)	2.8 (168)	0.148
Other opioids	18.8 (3)	14.1 (111)	0.486	18.7 (47)	12.5 (749)	**0.004**
Sedatives	43.8 (7)	26.4 (208)	0.121	40.9 (103)	28.5 (1704)	**<0.001**
Cocaine	12.5 (2)	5.3 (42)	0.217	11.1 (28)	13.8 (826)	0.221
Amphetamine	56.3 (9)	54.4 (429)	0.886	58.7 (148)	50.3 (3006)	**0.009**
Cannabis	43.8 (7)	38.1 (300)	0.643	62.3 (157)	53.2 (3180)	**0.004**
Injection drug use	56.3 (9)	48.2 (380)	0.525	59.5 (150)	39.6 (2366)	**<0.001**
*Mean number of substances used, last 30 days *	1.4	1.3	0.769	1.7	1.4	**<0.001**
*Main crime in index verdict*						
Violent crime	6.3 (1)	10.2 (80)	1.000	15.5 (39)	13.4 (799)	0.336
Property crime	6.3 (1)	15.6 (123)	0.489	23.4 (59)	21.9 (1308)	0.564
Drug crime	31.3 (5)	24.6 (194)	0.543	21.0 (53)	23.0 (1376)	0.463
Financial crime	0.0 (0)	6.2 (49)	0.616	2.4 (6)	3.9 (232)	0.224
*Lifetime history of psychiatric problems*						
Hospitalization	25.0 (4)	21.7 (171)	0.761	18.3 (46)	13.8 (825)	**0.046**
Suicide attempt	43.8 (7)	35.0 (276)	0.469	25.4 (64)	18.7 (1121)	**0.008**
Depression	68.8 (11)	62.6 (493)	0.612	47.2 (119)	49.4 (2954)	0.497
Anxiety	68.8 (11)	66.6 (525)	0.858	54.8 (138)	51.2 (3060)	0.265
Cognitive problems	62.5 (10)	52.7 (415)	0.435	54.0 (136)	51.6 (3083)	0.454
Hallucinations	12.5 (2)	13.2 (104)	1.000	11.9 (30)	12.8 (763)	0.689
Difficulty controlling violent behaviour	37.5 (6)	32.1 (253)	0.648	38.1 (96)	42.0 (2509)	0.223

**Table 3:** Men: mortality risk factors.

	Hazard ratio	95% confidence interval	*p* value
Mean age^*∗*^	1.026	1.013–1.040	**<0.001**
Country of birth, Nordic country	1.446	0.980–2.135	0.063
Chronic physical disease	1.025	0.789–1.332	0.851
Work for the last 3 yrs	0.781	0.588–1.038	0.088
*Lifetime history of substance abuse (>1 yr)*			
Binge drinking	1.001	0.768–1.304	0.995
Heroin^*∗*^	1.737	1.261–2.393	**0.001**
Other opioids	0.938	0.655–1.345	0.729
Sedatives^*∗*^	1.404	1.026-1.923	**0.034**
Amphetamine^*∗*^	0.684	0.491–0.953	**0.025**
Cannabis	1.067	0.791–1.439	0.670
Injection drug use^*∗*^	1.430	1.013–2.018	**0.042**
Mean number of substances used, last 30 days	1.040	0.922–1.172	0.524
*Lifetime history of psychiatric problems*			
Hospitalization	1.099	0.784–1.541	0.583
Suicide attempt	1.136	0.839–1.539	0.410

^*∗*^Variable significantly associated with the outcome variable.

**Table 4:** Characteristics of clients with and without return to the criminal justice system during follow-up.

	Women			Men		
	Clients relapsing, % (*n*)	Clients not relapsing, % (*n*)	*p* value	Clients relapsing, % (*n*)	Clients not relapsing, % (*n*)	*p* value
*N* =	*254*	*153*		*2599*	*1075*	
Mean age	36.15 years	37.27 years	0.258	33.03 years	33.28 years	0.256
Urban residence, large city	45.7 (116)	39.2 (60)	0.203	43.7 (1136)	49.6 (533)	**0.001**
Country of birth, Nordic country	96.1 (244)	92.2 (141)	0.091	84.6 (2198)	78.8 (847)	**<0.001**
Homelessness, last 30 days	34.3 (87)	23.5 (36)	**0.023**	25.2 (654)	14.5 (15)	**<0.001**
Client has children	65.7 (167)	68.6 (105)	0.550	50.8 (1320)	45.2 (486)	**0.002**
Chronic physical disease	65.0 (165)	52.9 (81)	**0.016**	47.9 (1244)	44.6 (479)	0.068
Client has worked for the last 3yrs	24.4 (62)	32.7 (50)	0.070	30.8 (801)	47.1 (506)	**<0.001**
Client has partner with substance abuse	53.9 (137)	38.6 (59)	**0.003**	13.3 (345)	9.8 (105)	**0.003**
*Lifetime history of substance abuse (>1 yr)*						
Binge drinking	47.6 (121)	49.0 (75)	0.787	45.4 (1181)	45.6 (490)	0.938
Heroin	29.5 (75)	17.6 (27)	**0.007**	23.9 (621)	16.0 (172)	**<0.001**
Methadone	9.4 (24)	2.0 (3)	**0.003**	3.6 (93)	2.9 (31)	0.289
Other opioids	15.4 (39)	13.7 (21)	0.653	15.3 (397)	13.9 (149)	0.273
Sedatives	37.8 (96)	32.0 (49)	0.239	37.2 (967)	31.7 (341)	**0.002**
Cocaine	7.9 (20)	5.2 (8)	0.307	16.6 (431)	19.1 (205)	0.070
Amphetamine	81.5 (207)	62.1 (95)	**<0.001**	67.6 (1757)	50.8 (546)	**<0.001**
Cannabis	53.5% (136)	42.5% (65)	**0.031**	64.3% (1672)	61.7 (663)	0.128
Injection drug use	79.1% (201)	56.9% (87)	**<0.001**	57.9% (1505)	35.2% (378)	**<0.001**
Mean number of substances used, last 30 days	2.0	1.5	**<0.001**	1.9	1,6	**<0.001**
*Main crime in index verdict*						
Violent crime	7.1 (18)	20.9 (32)	**<0.001**	14.4 (373)	20.8 (224)	**<0.001**
Property crime	32.7 (83)	15.0 (23)	**<0.001**	34.3 (891)	20.8 (224)	**<0.001**
Drug crime	37.4 (95)	43.1 (66)	0.252	27.7 (719)	38.4 (413)	**<0.001**
Financial crime	5.1 (13)	3.9 (6)	0.579	5.1 (132)	3.3 (35)	**0.016**
*Lifetime history of psychiatric problems*						
Hospitalization	23.2 (59)	15.0 (23)	**0.046**	16.6 (432)	12.7 (137)	**0.003**
Suicide attempt	32.7 (50)	39.4 (100)	0.175	22.5 (585)	19.3 (208)	**0.034**
Depression	59.5 (91)	60.2 (153)	0.880	50.6 (1315)	51.0 (548)	0.834
Anxiety	64.6 (164)	65.4 (100)	0.871	54.4 (1413)	53.0 (570)	0.457
Cognitive problems	53.9 (137)	56.2 (86)	0.656	57.7 (1500)	53.1 (571)	**0.011**
Hallucinations	16.1 (41)	16.3 (25)	0.958	14.2 (369)	12.0 (129)	0.077
Difficulty controlling violent behaviour	38.2 (97)	34.0 (52)	0.394	47.9 (1245)	39.2 (421)	**<0.001**

**Table 5:** Women: risk factors for criminal recidivism.

	Hazard ratio	95% confidence interval	*p* value
Homelessness, last 30 days	1.109	0.846–1.455	0.454
Chronic physical disease	1.193	0.911–1.561	0.200
Partner with substance abuse^*∗*^	1.320	1.022–1.704	**0.034**
*Lifetime history of substance abuse (>1 yr)*			
Heroin	0.992	0.722–1.363	0.959
Amphetamine	1.229	0.829–1.822	0.304
Cannabis	0.899	0.676–1.195	0.464
Injection drug use	1.327	0.902–1.953	0.151
Mean number of substances used, last 30 days	1.052	0.944–1.171	0.361
*Main crime in index verdict*			
Violent crime^*∗*^	0.525	0.316–0.873	**0.013**
Property crime^*∗*^	1.368	1.037–1.803	**0.027**
*Lifetime history of psychiatric problems*			
Hospitalization	1.214	0.901–1.635	0.202

^*∗*^Variable significantly associated with the outcome variable.

**Table 6:** Men: risk factors for criminal recidivism.

	Hazard ratio	95% confidence interval	*p* value
Urban residence, large city^*∗*^	0.912	0.843–0.987	**0.023**
Country of birth, Nordic country	1.060	0.946–1.188	0.317
Homelessness, last 30 days^*∗*^	1.163	1.059–1.276	**0.001**
Client has children	1.047	0.966–1.134	0.264
Work for the last 3 yrs^*∗*^	0.748	0.686–0.815	**<0.001**
Partner with substance abuse	1.018	0.907–1.142	0.766
*Lifetime history of substance abuse (>1 yr)*			
Heroin^*∗*^	1.147	1.036–1.269	**0.008**
Sedatives	0.960	0.876–1.053	0.390
Amphetamine^*∗*^	1.146	1.036–1.269	**0.008**
Injection drug use^*∗*^	1.461	1.326–1.610	**<0.001**
Mean number of substances used, last 30 days	1.008	0.971–1.046	0.686
*Main crime in index verdict*			
Violent crime^*∗*^	0.762	0.665–0.873	**<0.001**
Property crime^*∗*^	1.235	1.104–1.382	**<0.001**
Drug crime^*∗*^	0.815	0.724–0.917	**0.001**
Economical crime	1.151	0.948–1.398	0.155
*Lifetime history of psychiatric problems*			
Hospitalization	1.074	0.964–1.197	0.194
Suicide attempt	0.933	0.847–1.029	0.166
Cognitive problems	1.026	0.944–1.114	0.548
Difficulty controlling violent behaviour^*∗*^	1.215	1.119–1.320	**<0.001**

^*∗*^Variable significantly associated with the outcome variable.
